# Simple Epithelial Hypopharyngeal Cyst Presenting as Acute Airway Obstruction in a Three‐Year‐Old Child: A Case Report and Literature Review

**DOI:** 10.1002/ccr3.73666

**Published:** 2026-09-30

**Authors:** Fathalla Noori, Sohaib Salahat, Abdelrahman Salous, Thabet M. Thabet, Khaled Nazzal, Abdulhafeez Daghlas

**Affiliations:** ^1^ Department of Medicine, Faculty of Medicine and Health Sciences An‐Najah National University Nablus Palestine; ^2^ Department of Otolaryngology‐Head and Neck Surgery Rafidia Surgical Hospital Nablus Palestine

**Keywords:** airway obstruction, endoscopic excision, hypopharyngeal cyst, pediatric emergency, stridor

## Abstract

Hypopharyngeal cysts are a rare cause of pediatric upper airway obstruction and may present acutely, even in previously asymptomatic children. Early endoscopic evaluation is essential in children with stridor and unexplained airway compromise, as prompt surgical excision can be both diagnostic and curative.

## 1 Introduction

Hypopharyngeal cysts are uncommon, benign lesions occurring in the upper aerodigestive tract and represent an uncommon cause of airway obstruction in the pediatric population. They include a diverse range of congenital and acquired hypopharyngeal cysts, including retention cysts, ductal cysts, foregut duplication cysts, bronchogenic cysts, and other epithelial‐lined cysts [1, 2]. Although histologically benign, their location adjacent to the laryngeal inlet may result in significant morbidity from airway compromise, especially in young children with relatively narrow upper airways [3].

Clinical presentation differs widely with the lesion size and anatomical location. Small cysts are usually asymptomatic, but larger lesions can present with symptoms such as dysphagia, feeding difficulties, recurrent respiratory symptoms, stridor, voice changes, or acute respiratory distress [4]. Because symptoms are frequently nonspecific and the condition is rare, diagnosis may be delayed or missed initially. Sometimes, previously asymptomatic lesions become clinically manifest only after sudden enlargement or positional prolapse into the glottis, leading to potentially life‐threatening obstruction [2, 4].

Evaluation typically requires a combination of endoscopic assessment and cross‐sectional imaging. Flexible nasopharyngolaryngoscopy allows direct visualization of the lesion and evaluation of airway patency, while computed tomography (CT) and magnetic resonance imaging (MRI) help define the extent of disease and its relationship to the deep neck space [5, 6]. Surgical excision is the treatment of choice in most cases, and diagnosis is confirmed by histopathological examination [3]. Simple epithelial hypopharyngeal cysts in the pediatric population are exceedingly rare, with the literature consisting mainly of isolated case reports and small case series. As a result, the natural history, optimal management, and range of clinical presentations are not fully defined [1, 4].

We report a 3‐year‐old girl who presented with an acute upper airway presentation of a simple epithelial hypopharyngeal cyst. Unlike most previously reported pediatric hypopharyngeal cysts, which typically present with a subacute or chronic history of stridor, feeding difficulty, or recurrent respiratory symptoms, this case is notable for its acute, previously asymptomatic presentation with no antecedent airway history, requiring rapid diagnostic evaluation and emergent airway management. The case highlights the diagnostic challenges associated with this lesion, the importance of endoscopic and radiological evaluation in airway evaluation, and the good outcome achieved following complete transoral excision.

## 2 Case History and Examination

A 3‐year‐old female child with no significant past medical history, no previous surgical procedures, and no known drug allergies was brought to the emergency department by her parents with a sudden onset of breathing difficulty and throat pain. According to the caregivers, the child had been well the previous evening, but on the morning of presentation had developed a low‐grade fever, for which no documentation was available. There was no history suggestive of foreign body aspiration, no recurrent respiratory infections, no prior episodes of stridor or dysphonia, no dysphagia, and no preceding voice changes.

Upon initial assessment, the child appeared well and was alert and conscious. She was pink in color with no central or peripheral cyanosis. Vital signs were stable, with no evidence of hemodynamic compromise. The upper airway was patent at rest. A notable finding on dynamic examination was a translucent, smooth, bulging lesion that became visible in the posterior oropharynx during swallowing, suggesting a lesion with positional variability. Conventional oropharyngeal examination did not permit adequate characterization of its extent or origin.

## 3 Differential Diagnosis, Investigations, and Treatment

Flexible nasopharyngolaryngoscopy was subsequently performed and demonstrated a well‐defined, translucent, fluid‐filled cystic mass situated superior to the laryngeal inlet, causing partial narrowing of the supraglottic airway. The mucosal surface overlying the lesion appeared intact. The precise anatomical origin of the lesion, whether from the hypopharyngeal wall, the posterior surface of the epiglottis, or an adjacent mucosal fold, could not be definitively established at the time of initial endoscopic evaluation due to the child's age and limited cooperation (Figure 1).

**FIGURE 1:**
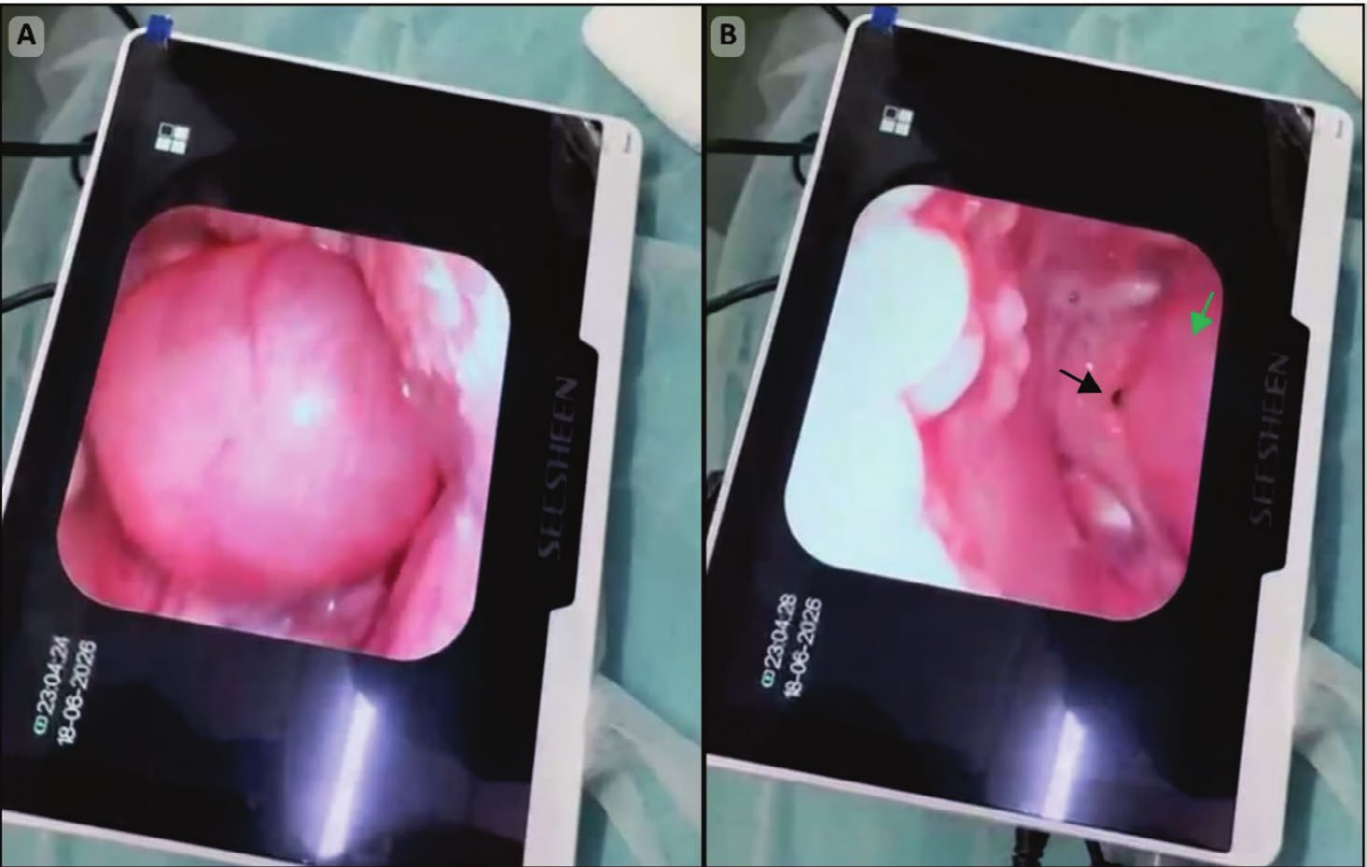
Flexible fiberoptic nasopharyngolaryngoscopic findings of the supraglottic cystic lesion. (A) View demonstrating a smooth, translucent, fluid‐filled mass located superior to the laryngeal inlet with an intact, non‐inflamed mucosal surface. (B) View demonstrating the relationship of the lesion to the vocal cords, with partial obscuration and narrowing of the supraglottic airway; preserved vocal cord mobility was observed on dynamic assessment. The mass and vocal cords are indicated by green and black arrows, respectively.

Contrast‐enhanced CT of the neck was performed and demonstrated a well‐defined, homogeneously hypodense, thin‐walled cystic lesion measuring approximately 2 cm in greatest diameter arising from the hypopharyngeal/supraglottic region. The lesion caused anterior displacement of the supraglottic structures with associated narrowing of the upper airway lumen. The lesion exhibited benign imaging characteristics, without internal enhancement, septations, solid components, or calcifications, with only minimal smooth peripheral wall enhancement. Multiplanar reformatted images confirmed the lesion's extent, its extrinsic relationship to the laryngeal framework, and its effect on the adjacent airway. No regional lymphadenopathy or osseous abnormalities were identified. The overall CT appearances were most consistent with a benign epithelial or retention cyst (Figure 2).

**FIGURE 2:**
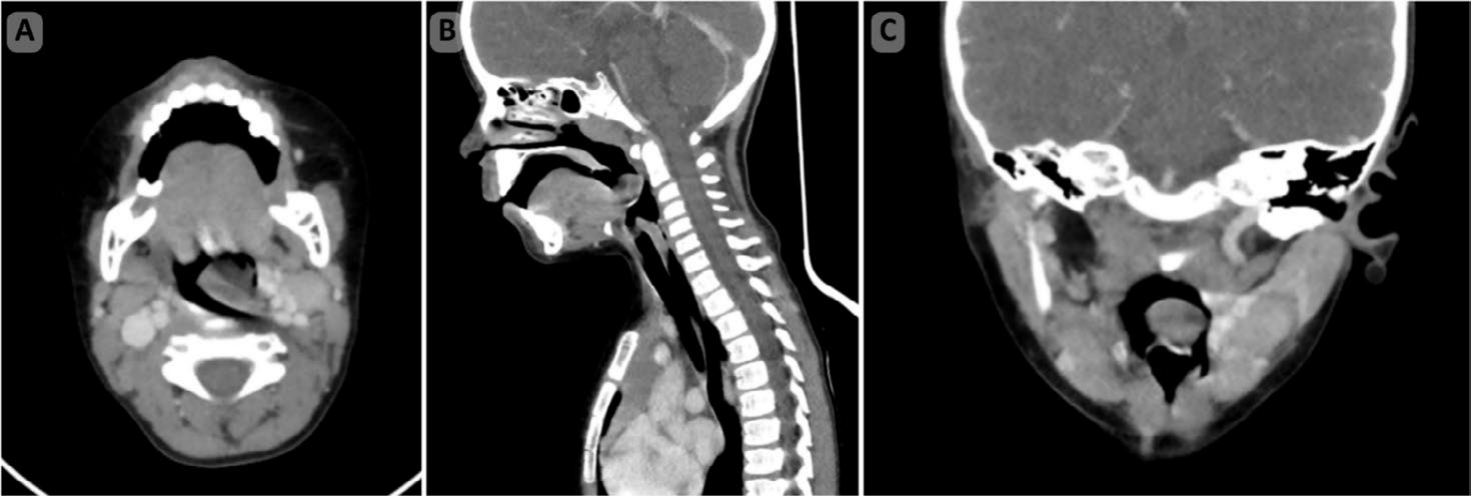
Contrast‐enhanced CT imaging demonstrating the cystic lesion in multiplanar views. (A) Axial CT image at the supraglottic level showing a well‐defined, thin‐walled, homogeneous hypodense cystic lesion occupying the posterior hypopharyngeal space, with anterior displacement of the supraglottic structures and resultant narrowing of the upper airway lumen. The lesion demonstrates no internal enhancement, septations, solid components, or calcifications, with minimal smooth peripheral wall enhancement. (B) Sagittal reformatted CT image illustrating the craniocaudal extent of the lesion and its relationship to the laryngeal inlet, demonstrating anterior displacement of the supraglottic tissues with preserved residual airway patency. (C) Coronal reformatted CT image demonstrating the superior–inferior extent of the lesion in relation to the hypopharynx and laryngeal inlet, confirming its extrinsic relationship to the laryngeal framework. No regional lymphadenopathy or osseous abnormality is identified.

Following multidisciplinary discussion between the pediatric emergency team and otolaryngology, the patient was admitted for close airway monitoring. Conservative measures were instituted, including placement of the patient in a lateral decubitus position to minimize gravitational prolapse of the lesion over the laryngeal inlet, strict avoidance of the fully supine position, and maintenance of nil per os (NPO) status from midnight. Pharmacological management consisted of intravenous dexamethasone to reduce peri‐lesional mucosal edema, nebulized adrenaline to attenuate airway mucosal swelling, and nebulized budesonide as an adjunctive anti‐inflammatory agent. The child remained hemodynamically stable throughout the observation period, with no deterioration in respiratory status.

Surgical excision was performed the following day under general anesthesia. The patient was positioned supine with the neck extended to optimize laryngoscopy access. Pharyngeal packing was placed to protect the lower airway and control oral secretions. Direct laryngoscopy revealed a hypopharyngeal cystic mass situated between the base of the tongue and the posterior tonsillar pillar region. The lesion was well encapsulated with a smooth outer surface and appeared fluid‐filled. Complete excision of the cystic lesion was accomplished using cold instruments without entry into the cyst cavity. The patient remained vitally stable throughout the procedure.

The excised specimen measured approximately 2.5 × 2 cm. Histopathological examination revealed a cyst wall composed of fibrovascular connective tissue with reactive inflammatory cell infiltrates. The cyst lining comprised non‐keratinized stratified squamous epithelium in some areas and respiratory‐type columnar epithelium in others, consistent with a simple epithelial cyst of mixed mucosal lineage. No cytological atypia, dysplasia, or features of malignancy were identified. Cytological preparations from the cyst fluid were hypocellular and demonstrated reactive inflammatory cells and scattered squamous epithelial cells, with no atypical or malignant cells. The combined histological and cytological findings established the definitive diagnosis of a simple epithelial hypopharyngeal cyst with no evidence of malignancy.

The postoperative course was uneventful. The child was fully conscious and clinically stable in the immediate recovery period. The upper airway remained patent, and the oral cavity was clear. The patient tolerated oral fluid intake without difficulty and subsequently commenced on a soft diet. She was discharged home on a course of oral antibiotics. Outpatient otolaryngology follow‐up was arranged in the clinic 2 weeks post‐discharge, at which time the child was well with no residual respiratory or swallowing symptoms. A subsequent follow‐up visit was conducted 5 months after the initial discharge, at which the child remained asymptomatic, with no evidence of recurrence or other complications.

## 4 Conclusion

Simple epithelial hypopharyngeal cysts are rare in children but may present with acute, life‐threatening airway obstruction without antecedent symptoms. This case illustrates the diagnostic challenge posed by such lesions and underscores the central roles of flexible nasopharyngolaryngoscopy and contrast‐enhanced CT in rapid evaluation and operative planning. Conservative airway management, including positional measures and pharmacological agents, may temporize acute obstruction and permit elective rather than emergency surgery. Complete transoral excision under general anesthesia is safe, technically feasible, and curative in the majority of cases. Histopathological analysis is essential to confirm a benign disease and guide subsequent follow‐up. Clinicians managing pediatric airway emergencies should include hypopharyngeal cysts in their differential diagnosis, even in the absence of a prior suggestive history.

## 5 Discussion

### 5.1 Clinical Presentation and Pathophysiology

Laryngeal and hypopharyngeal cysts in children are uncommon but clinically significant lesions. Their prevalence is difficult to ascertain precisely, given the paucity of large series. However, case reports and small case series have appeared with sufficient frequency to suggest that hypopharyngeal cysts are the likely etiology in a meaningful proportion of pediatric patients presenting with otherwise unexplained upper airway obstruction [5, 7]. The present case is notable for the acuity of presentation: a previously well 3‐year‐old with no antecedent airway history who developed acute respiratory distress without any recognized precipitant beyond a transient low‐grade fever.

The anatomical basis for symptom severity in hypopharyngeal cysts relates directly to the proximity of the lesion to the laryngeal inlet. Lesions arising in or near the vallecular or supraglottic region are particularly prone to causing obstruction because even modest enlargement encroaches on the narrow pediatric supraglottic airway, compressing the residual airway lumen or prolapsing over the laryngeal inlet, particularly when the child assumes a supine position [8, 9]. This mechanism, positional worsening of obstruction in the supine compared with the lateral or prone position, is well documented in the vallecular cyst literature and was observed in the present case, where positional management formed a critical component of initial conservative therapy. The child's lateral decubitus positioning was not merely symptomatic but mechanistically sound, as it reduced the gravitational vector acting on the lesion toward the glottis.

The sudden onset of respiratory distress in this patient, in the context of an undocumented low‐grade fever, raises the important question of whether an acute inflammatory event contributed to rapid lesion enlargement. It has been postulated that retention cysts may enlarge acutely due to inflammatory obstruction of their drainage orifice, a mechanism broadly analogous to that of acutely infected epidermal inclusion cysts in soft tissues [3]. This would explain why the lesion had not previously come to clinical attention despite presumably existing for some time. Such a presentation pattern, quiescent lesion with acute decompensation, is represented in the comparative literature [5, 10] and should heighten clinical suspicion for a cystic etiology in children with apparent croup or acute epiglottitis who do not respond as expected to conventional pharmacotherapy. Relevant articles were identified through targeted searches of PubMed for recent case reports and case series on pediatric hypopharyngeal, supraglottic, and vallecular cysts, supplemented by manual screening of reference lists from retrieved articles. Priority was given to recent publications directly relevant to airway obstruction, diagnosis, and surgical management in this patient population (Table 1).

**TABLE 1:** Published pediatric cases of hypopharyngeal cysts presenting with airway symptoms.

Author (year)	Age/Sex	Clinical presentation	Lesion location	Histopathology	Treatment
Roy et al. (2011) [5]	5 y/M	Recurrent aspiration pneumonia, dysphagia, obstructed breathing, weak voice	Posterior hypopharyngeal wall	Foregut duplication cyst with gastric‐type epithelium	Transoral complete excision
Baranski et al. (2022) [10]	4 mo/F	Stridor, feeding difficulty, respiratory distress	Hypopharynx with extension to upper cervical esophagus	Duplication cyst lined by respiratory and squamous epithelium with smooth and striated muscle fibers	Complete surgical excision
Rehman et al. (2026) [11]	2 mo/F	Progressive inspiratory stridor, respiratory distress, vomiting, and fever over one month.	Vallecula, between tongue base and epiglottis, compressing aryepiglottic folds and arytenoids	Benign vallecular cyst	Tracheostomy followed by cyst excision
Present case	3 y/F	Acute airway obstruction	Hypopharynx	Simple epithelial cyst lined by non‐keratinized squamous and respiratory epithelium without a muscular wall	Endoscopic excision

### 5.2 Diagnostic Evaluation

Flexible nasopharyngolaryngoscopy was the pivotal diagnostic procedure in this case. In children presenting with stridor, dyspnea, or unexplained upper airway obstruction, flexible endoscopy provides direct and dynamic visualization of the entire upper aerodigestive tract with minimal invasiveness [5, 12]. The translucent, smooth‐walled cystic nature of the lesion was immediately apparent on endoscopy and largely excluded inflammatory, vascular, and solid neoplastic etiologies at the bedside. However, because the precise point of attachment of the lesion could not be determined from flexible endoscopy alone, an acknowledged limitation in a young, anxious child, cross‐sectional imaging was essential for operative planning.

Contrast‐enhanced CT of the neck provided complementary and decisive information in three respects. First, it confirmed the benign cystic nature of the lesion through demonstration of homogeneous hypodensity, smooth thin walls, absence of internal septations, and minimal peripheral enhancement without central enhancement of the cyst contents. Second, it delineated the lesion's relationships to adjacent structures, including its superior and inferior extent and the degree of airway compromise, enabling a rational decision about the timing and urgency of surgical intervention. Third, it excluded alternative diagnoses with potentially more complex surgical implications, including vascular malformation, thyroglossal duct cyst, and malignancy [13]. These three functions, lesion characterization, anatomical mapping, and differential diagnosis exclusion, justify CT imaging as a routine component of the workup of hypopharyngeal cysts in children, provided the patient is stable enough to safely undergo the examination.

### 5.3 Differential Diagnosis

The differential diagnosis of a cystic hypopharyngeal mass in a child includes several entities distinguished primarily by location and imaging characteristics. Vallecular cysts arise from the base of tongue/vallecula and typically present in neonates and infants [8, 9, 11]; the present lesion's more posterior, hypopharyngeal location excluded this diagnosis. Saccular cysts and laryngoceles arise from the laryngeal saccule and present with hoarseness, which was absent here [9, 14]. Thyroglossal duct cysts present as midline neck masses that move with tongue protrusion rather than as intraluminal pharyngeal masses [15, 16]. Lymphatic malformations are typically multiloculated and infiltrative on CT [17], unlike the unilocular lesion seen here. Congenital foregut duplication cysts and branchial cleft anomalies were considered but required histopathological exclusion [1, 18] (Table 2).

**TABLE 2:** Differential diagnosis and distinguishing features in this case.

Entity	Typical features	Distinguishing finding in this case
Vallecular cyst	Arises from the vallecula at the tongue base; most common pediatric supraglottic cyst; typically presents with stridor in neonates/infants	Excluded by the more posterior and lateral lesion location on CT and at surgery
Saccular cyst	Arises from the laryngeal saccule, within the laryngeal ventricle; presents with hoarseness and stridor	Excluded by absence of dysphonia and CT localization above the laryngeal inlet
Laryngocele (internal/external)	External laryngocele communicates with the laryngeal saccule as a neck mass; internal laryngocele remains confined within the laryngeal framework	Did not correspond to the imaging or intraoperative findings
Thyroglossal duct cyst	Most common congenital midline cervical cyst; presents as a midline neck mass at or below the hyoid, moving with tongue protrusion	Not an intraluminal hypopharyngeal mass; no midline neck mass identified
Lymphatic malformation (cystic hygroma)	Low‐flow vascular malformation; tends to be multiloculated, infiltrative, and poorly marginated on CT	Excluded by the unilocular, well‐defined, non‐infiltrative appearance
Foregut duplication cyst	Rare; may occasionally present in the hypopharyngeal region with unusual features	Considered and definitively excluded by histopathological examination
Branchial cleft cyst/pyriform sinus fistula	Arises near the piriform sinus	Excluded by anatomical location and intraoperative findings

### 5.4 Histopathology and Management

Histopathological confirmation is indispensable for clarifying cyst lineage and excluding dysplasia and malignancy. The present specimen demonstrated a cyst wall lined by a combination of non‐keratinized stratified squamous epithelium and respiratory columnar epithelium, consistent with a simple epithelial cyst arising from the mucosal lining of the hypopharynx, where transitional zones between squamous and respiratory epithelium are physiologically present. The reactive inflammatory infiltrate within the cyst wall corroborated the hypothesis of acute inflammatory enlargement as a precipitant of the presentation. The absence of dysplasia was reassuring and, together with complete excision, eliminated the need for adjuvant therapy or enhanced surveillance intervals beyond routine follow‐up.

Surgical excision by the transoral route, performed under direct laryngoscopy with the patient under general anesthesia, is the accepted standard of care for hypopharyngeal cysts in children [19, 20]. The goals of surgery are complete removal of the cyst, ideally including the entire wall to minimize the risk of recurrence, and preservation of adjacent mucosal integrity. In the present case, complete transoral excision was feasible given the lesion's size and location, and the procedure was uncomplicated. The outcome was excellent, consistent with published reports in which complete excision uniformly results in symptom resolution and a low recurrence rate.

## 6 Limitations

Several limitations of this report merit acknowledgement. The precise developmental origin of the lesion, whether arising from minor salivary gland tissue, from respiratory epithelial rests, or from obstructed mucosal glands, cannot be established with certainty based on the histopathological findings described, as the specimen demonstrated both squamous and respiratory epithelial lining without a clearly dominant component. Although follow‐up to five months confirms the durability of the clinical response and shows no evidence of recurrence, this duration remains insufficient to permit comment on very long‐term recurrence rates for this specific lesion. Finally, as acknowledged in the literature review, the published evidence base for pediatric hypopharyngeal cysts comprises predominantly isolated case reports rather than controlled series, which limits the extent to which generalizable recommendations can be derived.

## Author Contributions

**Fathalla Noori:** writing – original draft, writing – review and editing, validation. **Sohaib Salahat:** writing – original draft, writing – review and editing, validation. **Abdelrahman Salous:** writing – original draft, writing – review and editing. **Thabet M. Thabet:** writing – original draft, writing – review and editing. **Khaled Nazzal:** visualization, supervision, data curation. **Abdulhafeez Daghlas:** visualization, supervision.

## Funding

The authors have nothing to report.

## Consent

Written informed consent was obtained from the patient's parents/legal guardians for publication of this case report and any accompanying clinical images, in accordance with the ethical standards of the institution and the Declaration of Helsinki.

## Conflicts of Interest

The authors declare no conflicts of interest.

## Data Availability

Data sharing not applicable to this article as no datasets were generated or analysed during the current study.
